# Confidence-Aware Severity Assessment of Lung Disease from Chest X-Rays Using Deep Neural Network on a Multi-Reader Dataset

**DOI:** 10.1007/s10278-024-01151-5

**Published:** 2024-08-20

**Authors:** Mohammadreza Zandehshahvar, Marly van Assen, Eun Kim, Yashar Kiarashi, Vikranth Keerthipati, Giovanni Tessarin, Emanuele Muscogiuri, Arthur E. Stillman, Peter Filev, Amir H. Davarpanah, Eugene A. Berkowitz, Stefan Tigges, Scott J. Lee, Brianna L. Vey, Carlo De Cecco, Ali Adibi

**Affiliations:** 1 School of Electrical and Computer Engineering, Georgia Institute of Technology, Atlanta, USA; 2 Department of Radiology and Imaging Sciences, Emory School of Medicine, Emory University, Atlanta, USA; 3 Department of Biomedical Informatics, Emory School of Medicine, Emory University, Atlanta, USA

**Keywords:** Lung disease, Severity, Deep learning, Confidence-aware prediction, Uncertainty

## Abstract

In this study, we present a method based on Monte Carlo Dropout (MCD) as Bayesian neural network (BNN) approximation for confidence-aware severity classification of lung diseases in COVID-19 patients using chest X-rays (CXRs). Trained and tested on 1208 CXRs from Hospital 1 in the USA, the model categorizes severity into four levels (i.e., normal, mild, moderate, and severe) based on lung consolidation and opacity. Severity labels, determined by the median consensus of five radiologists, serve as the reference standard. The model’s performance is internally validated against evaluations from an additional radiologist and two residents that were excluded from the median. The performance of the model is further evaluated on additional internal and external datasets comprising 2200 CXRs from the same hospital and 1300 CXRs from Hospital 2 in South Korea. The model achieves an average area under the curve (AUC) of 0.94 ± 0.01 across all classes in the primary dataset, surpassing human readers in each severity class and achieves a higher Kendall correlation coefficient (KCC) of 0.80 ± 0.03. The performance of the model is consistent across varied datasets, highlighting its generalization. A key aspect of the model is its predictive uncertainty (PU), which is inversely related to the level of agreement among radiologists, particularly in mild and moderate cases. The study concludes that the model outperforms human readers in severity assessment and maintains consistent accuracy across diverse datasets. Its ability to provide confidence measures in predictions is pivotal for potential clinical use, underscoring the BNN’s role in enhancing diagnostic precision in lung disease analysis through CXR.

## Introduction

Lung disease is a leading cause of global mortality, emphasizing the importance of early diagnosis for effective treatment and prevention of permanent lung damage. Chest X-rays (CXRs) are commonly used for diagnosing and assessing the severity of lung diseases, including pneumonia, cancer, and chronic obstructive pulmonary disease, due to their accessibility, cost-effectiveness, and general anatomical orientation [1]. However, the increasing volume of imaging data and the high variability among radiologists in severity classification [2–6] pose challenges for timely diagnosis. Therefore, there is a growing need for automated and reliable approaches that can assist radiologists to reduce their workload, facilitate early diagnosis, and guide treatment decisions.

Artificial intelligence (AI), particularly deep learning (DL) models, has shown great promise in lung disease diagnosis using chest radiographs [7–10]. These methods also have been of great interest during the pandemic for diagnosis of pulmonary diseases [11–13]. Recent works on utilizing DL models demonstrated comparable or even superior performance compared to individual readers [14–17]. However, the availability of test kits shifted the focus towards severity assessment and prognosis of the disease [18–23].

Performance and generalization of DL models heavily depend on the quality and quantity of training data, and a multi-reader dataset is necessary for reliable training and evaluation. Despite the development of numerous AI algorithms, their clinical implementation has been hindered by the lack of transparency and reliability in their predictions [24, 25]. An ideal tool should provide uncertainty estimates to aid in interpretation and identify cases where predictions should be viewed with caution.

Bayesian neural networks (BNNs) offer a potential solution for reliable assessment of medical images, providing uncertainty estimates for predictions [26, 27]. Unlike regular neural networks, BNNs model the probability distribution of network weights, allowing for quantification of prediction uncertainty. This is particularly valuable in medical applications where incorrect predictions can have significant consequences. Integrating BNNs into AI-assisted tools would enable human readers to disregard predictions when model uncertainty is high.

This study introduces a new approach for severity assessment of lung diseases using CXRs based on Monte Carlo Dropout (MCD) as BNN approximation. The study focuses on the severity assessment of COVID-19 pneumonia without sacrificing generality, due to its significance and availability of data. Our proposed model is designed to provide the severity class along with prediction uncertainty and saliency maps to enable better interpretation and reliability. We compare the performance of our model with human readers and conduct additional internal and external evaluations to assess its reliability and effectiveness.

## Materials and Methods

### Data Collection and Acquisition

Consecutive adult patients (≥ 18 years old) who were diagnosed with COVID-19 using real-time reverse transcriptase-polymerase chain reaction (RT-PCR) and underwent diagnostic CXR from a hospital in the US and a hospital in South Korea were included in this study. Three datasets were created in this study for training and performance validation of the model. Dataset 1 was created by selecting patients from Hospital 1 (USA) seen between 01/30/2020 and 04/30/2020 and Dataset 2 from patients seen between 05/01/2020 and 11/05/2020, also from Hospital 1 (USA). Dataset 3 consisted of a consecutive cohort who underwent diagnostic CXR between 01/01/2020 and 06/01/2021 from Hospital 2 located in South Korea. Dataset 1 was used for training and testing the performance of the model. Additional internal validation was performed using Dataset 2, and Dataset 3 was used for external validation.

### Multi-Reader Study

The training set from Dataset 1 was labelled by six radiologists and two residents. The reference standard was considered as the median label of five radiologists (Rads. 1–5, selected randomly from the six radiologists) and the three other readers (Rad. 6, Res. 1 and 2) were kept out of the median for comparison with the AI model. For additional internal and external validation, Dataset 2 and Dataset 3 were labeled by three expert readers, and a collective label was assigned according to majority vote. All CXRs were classified according to the following guidelines:

1. Normal: no clear sign of disease exists in the lung regions.
2. Mild: opacification in the mid and lower lung zones; patchy and often peripheral in nature involving less than 25% of the most severely affected lung.
3. Moderate: bilateral opacification involving the peripheral mid and lower lung zones with approximately ≥ 25% and ≤ 50% involvement of the most severely affected lung.
4. Severe: patchy bilateral opacification with more than 50% involvement of the most severely affected lung.

All readers used the same guidelines and were provided with three examples for each class prior to labeling. The datasets were labeled by the readers independently with our in-house designed labeling tool, which randomizes the CXRs and allows the user to adjust the size of the images for better visualization. The readers did not have any information about the patients or other readers’ decisions and their assessment solely depended on the CXRs.

### DL Model for Confidence-Aware Severity Assessment of COVID-19 from CXRs

**Fig. 1:**
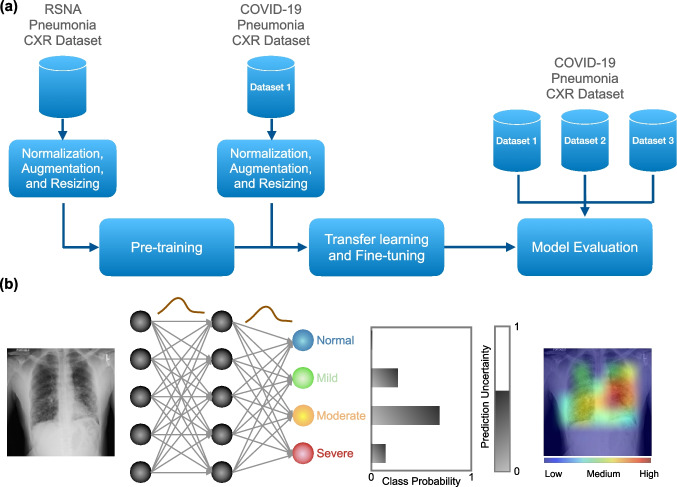
**a** Training and evaluation diagram of the model for severity assessment of the disease. The model was pre-trained on the RSNA CXR dataset, and transfer learning was used to fine tune the model for COVID-19 pneumonia severity classification over Dataset 1. Internal and external evaluations were performed using test data from Dataset 1, Dataset 2, and Dataset 3. **b** Schematic of the model for an example input CXR. The model predicts the severity and outputs the class probabilities, uncertainty of the prediction, and saliency map to highlight the lung regions that contribute the most in the decision

In our study, we implemented a confidence-aware DL model  to classify the severity of lung diseases from CXRs and simultaneously provide predictive uncertainty (PU) estimates. Implementing BNNs traditionally involves complex variational inference techniques that can be computationally intensive and difficult to tune. In contrast, our approach utilizes the MCD method [29], which simplifies the training process by integrating dropout layers not only during training but also during prediction. This dual use of dropout mimics Bayesian posterior inference, thus providing a computationally efficient and scalable solution to approximate Bayesian inference. The architecture of our model is an adaptation of the VGG-16 [28], consisting of five blocks of convolutional layers. Each convolutional layer is followed by max pooling and a dropout layer with a 0.25 dropout rate, which remains active during training to mitigate overfitting. The architecture culminates in a global average pooling layer, followed by a fully connected layer with 256 nodes and ReLU activation functions. The final layer comprises four neurons with SoftMax, each corresponding to a distinct severity class of the lung disease. Figure 1 shows the training and evaluation diagram of our model. The model was initially pre-trained on the RSNA (Radiological Society of North America) pneumonia dataset, which includes CXRs categorized as normal and pneumonia. Subsequently, the pre-trained weights were transferred for further training and fine-tuning on Dataset 1, focusing on COVID-19 pneumonia severity classification. During the training, each CXR was normalized to zero mean and unity standard deviation, and resized to 224 × 224 pixels. To enhance the model’s robustness to variations in the data, we employed a series of data augmentation techniques. Specifically, we used height and width shift ranges of 0.3, a rotation range of 8°, a shear range of 0.3, a zoom range of 0.3, and nearest fill mode, with sample-wise centering.

Dataset 1’s reference labels were based on the median consensus of five radiologists. To assess model performance, we compared it against the evaluations of three additional annotators (Radiologist 6, Resident 1, and Resident 2). We employed a bootstrapping method to create 20 separate training and test sets, ensuring the robustness and consistency of our results. Each round involved randomly dividing the dataset into training (83%) and test (17%) sets. During training, data augmentation methods including random rotation, zoom, shear, and horizontal and vertical shifts were applied. The network optimization used the Adam optimizer with a categorical cross-entropy loss function. The training was conducted in two phases. Initially, the convolutional layers were frozen, and only the fully connected layers were trained for 10 epochs at a learning rate of 1e-3 and a batch size of 32. In the second phase, all layers were unfrozen, the learning rate was reduced to 1e-4, and adaptive learning rate adjustments and early stopping (based on validation loss with a patience of 3) were employed. The entire model was implemented using TensorFlow Keras in Python, with computations performed on an NVIDIA RTX 2070 GPU.

The model’s performance was evaluated using the receiver operating characteristic curve (ROC) and area under the curve (AUC) for each severity class, with the mean and standard deviation reported. Additionally, we utilized the Kendall correlation coefficient to compare our model’s performance with human readers. Furthermore, we used Grad-CAM visualizations [30] to ensure that the model’s focus was appropriately on lung regions and not distracted by extraneous image elements. The model was further tested using Dataset 2 and Dataset 3 for additional internal and external validation, respectively. These datasets provided a comprehensive framework to assess the model’s generalizability and effectiveness in varied clinical settings.

To provide uncertainty of the model over the test data, we use PU which is a critical metric that quantifies the confidence of a model in its predictions in classification tasks. It is defined as the entropy of the model’s SoftMax output distribution for a given input. This entropy measures the spread of the predicted probabilities across different classes, with higher entropy indicating less certainty. For an unseen input ${x}^{*}$, the PU of the trained model using training dataset $D$ is as follows:

$$\mathrm H\left(\hat{\mathrm y}\vert\mathrm x^\ast,\mathrm D\right)=$$

1 $$-\sum_cp\left(\hat{y}=c\vert x^\ast,\;D\right)\;log\;p\left(\hat{y}=c\vert x^\ast,\;D\right)$$

This equation considers the entropy across all classes $c$, where $p(\widehat{y}=c|{x}^{*}, D)$ is the model’s predicted probability for class $c$ given the input ${x}^{*}$ and the training data $D$. Now, expanding on $p(\widehat{y}=c|{x}^{*}, D)$ and considering model’s weights $w$, Eq. (1) can be written as follows:

$$H(\widehat{y}|{x}^{*}, D)=$$

2 $$-\sum_c(\int\;p\left(\hat {y}\vert x^\ast,\;w\right)p\left(w\vert D\right)dw)\;(\log\;\int\;p\left(\hat {y}\vert x^\ast,\;w\right)p(w\vert D)dw)$$

Computing Eq. (2) is empirically infeasible, and we can approximate the true posterior $p\left(w|D\right)$ with a variational distribution $q(w)$. Thus, it simplifies to the following:

$$H(\widehat{y}|{x}^{*}, D)\approx$$

3 $$-\sum_c(\int\;p\left(\widehat y\vert x^\ast,\;w\right)q\left(w\right)dw)\;(\log\;\int\;p\left(\widehat y\vert x^\ast,\;w\right)q(w)dw)$$

Finally, we can approximate the integral with summation through $K$ steps of Monte Carlo sampling the weights ${w}_{k}$ from the variational distribution $q(w)$ as follows:

$$H(\widehat{y}|{x}^{*}, D)\approx$$

4 $$-\sum_c(\frac1K\sum_{k=1}^Kp(\hat {y}=c\vert x^\ast,\;W_k))\;(\log\frac1k\sum_{k=1}^Kp(\hat {y}=c\vert x^\ast,\;W_k)\;)$$

This final approximation provides a practical method for estimating predictive uncertainty, capturing the spread of the model’s predictions across different classes for a given input ${x}^{*}$. It is particularly valuable in high-stakes scenarios like medical diagnosis, where understanding the confidence level of predictions is crucial. In this work, the PU for each prediction was computed using Eq. (4) with $K=$ 100 steps of MCD [29].

## Results

### Dataset Characteristics

Dataset 1 included 1208 CXRs from 388 patients with confirmed RT-PCR within 4 weeks of the time of CXRs at Hospital 1 in the USA. The mean age of the patients in this dataset was 63 years (range, 50–74 years) with 50% of the patients being male. Dataset 2 included 2200 CXRs from 270 patients in Hospital 1 in the USA with a mean age of 61 years (range, 47–72 years) and 44.8% (*n* = 121) male patients. Dataset 3 included of 1219 CXRs from 266 patients with a mean age of 64 years (range, 44–84 years) and 43.6% male patients. Table 1 shows the patient characteristics and the detailed information for each dataset.

**Table 1:** Statistics and patient characteristics for Dataset 1 from Hospital 1 (USA), Dataset 2 from Hospital 1 (USA), and Dataset 3 from Hospital 2 (South Korea)

	*Dataset 1*	*Dataset 2*	*Dataset 3*
	*N* = 388	*N* = 270	*N* = 266
*Age*	62 $\pm$ 12	61 $\pm$ 13	64 $\pm$ 20
*Gender*	49.7% male (*n* = 193)	44.8% male (*n* = 121)	43.6% male (*n* = 116)
*BMI*	31.3 (25.9–35.7)	30.7 (25.9–36.1)	Not available

Datasets 1 and 2, which are from the same hospital, had similar severity class distribution with more cases from the higher severity classes, while Dataset 3 from Korea included more cases with lower severity scores. The number and percentage of CXRs for each severity class is presented in Table 2. Among 1208 CXRs from Dataset 1, the readers classified 18% of the data unanimously, 59% with one class difference, 21% with maximum of two class difference, and only 2% with maximum of three class difference. For Dataset 2, from 2200 CXRs, 46.79% were classified unanimously, 47.59% with one class difference, 5.42% with maximum of two class difference, and only 0.19% with three class difference. For Dataset 3, among 1219 CXRs, 57.83% were classified unanimously by the readers, 40.94% with one class difference, 1.07% with maximum of two class difference, and 0.16% with three class difference. Those that are classified with maximum of three class difference by the readers are mainly CXRs with low quality and/or windowing issue. More information regarding the reader variability can be found in [6].

**Table 2:** Severity class distribution for Dataset 1 from Hospital 1 (USA), Dataset 2 from Hospital 1 (USA), and Dataset 3 from Hospital 2 (South Korea)

Dataset/severity class	*Normal*	*Mild*	*Moderate*	*Severe*
Dataset 1	147 (12.7%)	358 (29.64%)	316 (29.16%)	387 (32.03%)
*Dataset 2*	156 (7.36%)	641 (30.24%)	509 (24.01%)	814 (38.40%)
*Dataset 3*	425 (34.87%)	461 (37.81%)	189 (15.50%)	144 (11.81%)

### Model Performance Evaluation

**Fig. 2:**
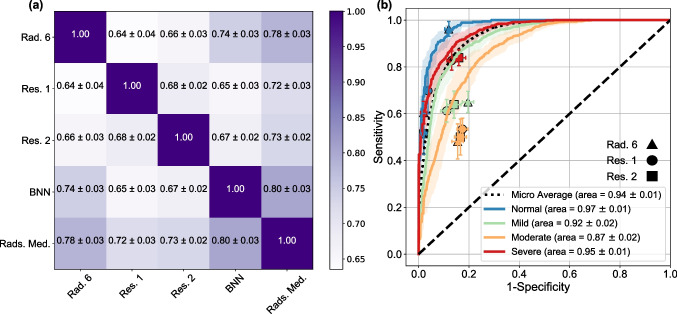
Performance of the trained DL model over test data from Dataset 1. The evaluation was performed using the bootstrap sampling method, and the mean and standard deviation of each performance metric are provided. **a** KCC for the model versus radiologists 6 (Rad. 6), resident 1 (Res. 1), and resident 2 (Res. 2) compared to the median of the five radiologists (Rads. Med.) as the reference standard. **b** The ROC of the model, the AUC, and the performance of the human readers over the test data

The model was trained over a random subset of Dataset 1 that included 1000 CXRs (83%) and kept the remaining data for testing the performance for internal validation (208 CXRS, 17%). As shown in Fig. 2(a), the model had higher agreement with the baseline (i.e., the median severity class labeled by five expert radiologists) compared to the human readers. The KCC between the output of the model and the median of expert ratings (Rads. Med.) was 0.80 $\pm$ 0.03; KCC was 0.78 $\pm$ 0.03, 0.72 $\pm$ 0.03, and 0.73 $\pm$ 0.02 for radiologist 6 (Rad. 6), resident 1 (Res. 1), and resident 2 (Res. 2), respectively. ROC of the model for different severity classes is presented in Fig. 2(b). The model had higher performance compared to the individual readers over different severity classes and significantly outperformed the expert readers over the moderate and mild classes (as shown in Fig. 2(b)), the ROCs of the model for all classes are on top of the operating points of the human readers). The AUC of the model was 0.97 $\pm$ 0.01, 0.92 $\pm$ 0.02, 0.87 $\pm$ 0.02, 0.95 $\pm$ 0.01, for the normal, mild, moderate, and sever classes, respectively. The overall AUC of the model was 0.94 $\pm$ 0.01.

**Fig. 3:**
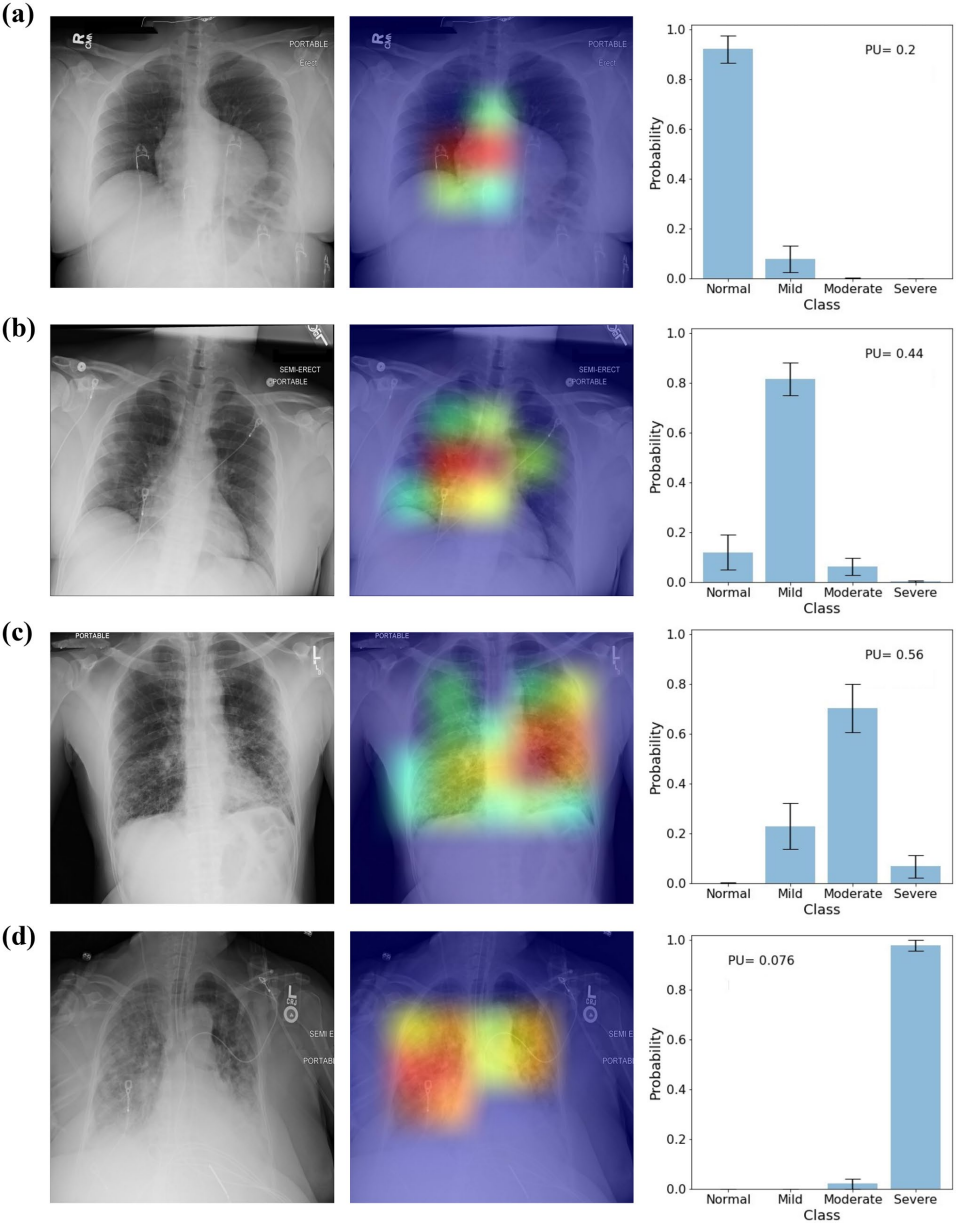
Examples of input CXRs and the corresponding saliency maps, class probabilities, and PU and EU for **a** normal, **b** mild, **c** moderate, and **d** severe classes of COVID-19 pneumonia

Some examples of the CXRs from the test set and the outputs of the model are shown in Fig. 3a–d. For an input CXR, the model provides the saliency map, which highlights regions in the image that contribute the most to the decision-making process, mean prediction probabilities and the standard deviations for each severity class, and the PU. The model correctly classified the CXRs in Fig. 3(a–d), and the saliency maps were focused on the lung regions and were not distracted by the pacemakers and artifacts in the images. The PU for the CXRs were 0.2, 0.44, 0.56, and 0.076, and they are shown as insets on the bar plots.

#### Additional Internal and External Validation

**Fig. 4:**
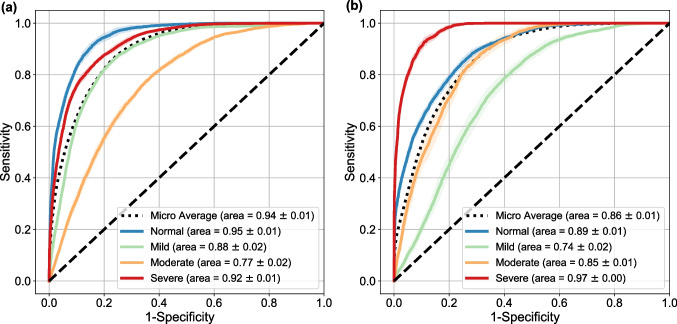
Additional internal and external validation of the model. ROC curves and AUC for the model based on data from **a** Dataset 2 and **b** Dataset 3

Figure 4(a) shows the ROC of the model for the additional internal validation analysis on Dataset 2 with overall AUC of 0.94 $\pm$ 0.01 and AUC of 0.95 $\pm$ 0.01, 0.88 $\pm$ 0.02, 0.77 $\pm$ 0.02, 0.92 $\pm$ 0.01 for the normal, mild, moderate, and severe classes. The overall AUC of the model on Dataset 3 for external validation was 0.86 $\pm$ 0.01 and the AUC was 0.89 $\pm$ 0.01, 0.74 $\pm$ 0.01, 0.85 $\pm$ 0.02, 0.97 $\pm$ 0.00 for the normal, mild, moderate, and severe classes, respectively, as shown in Fig. 4(b).

#### Predictive Uncertainty

**Fig. 5:**
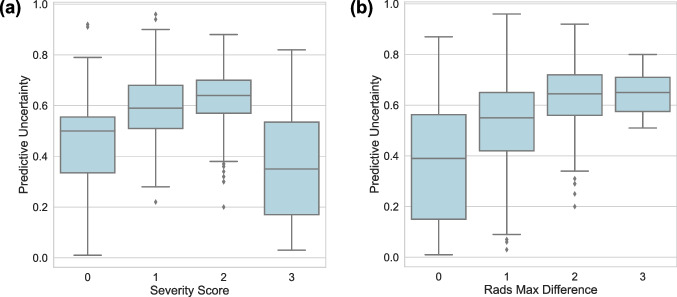
PU of the model versus **a** severity class of the CXRs and **b** maximum difference among the human readers over the internal test data

**Fig. 6:**
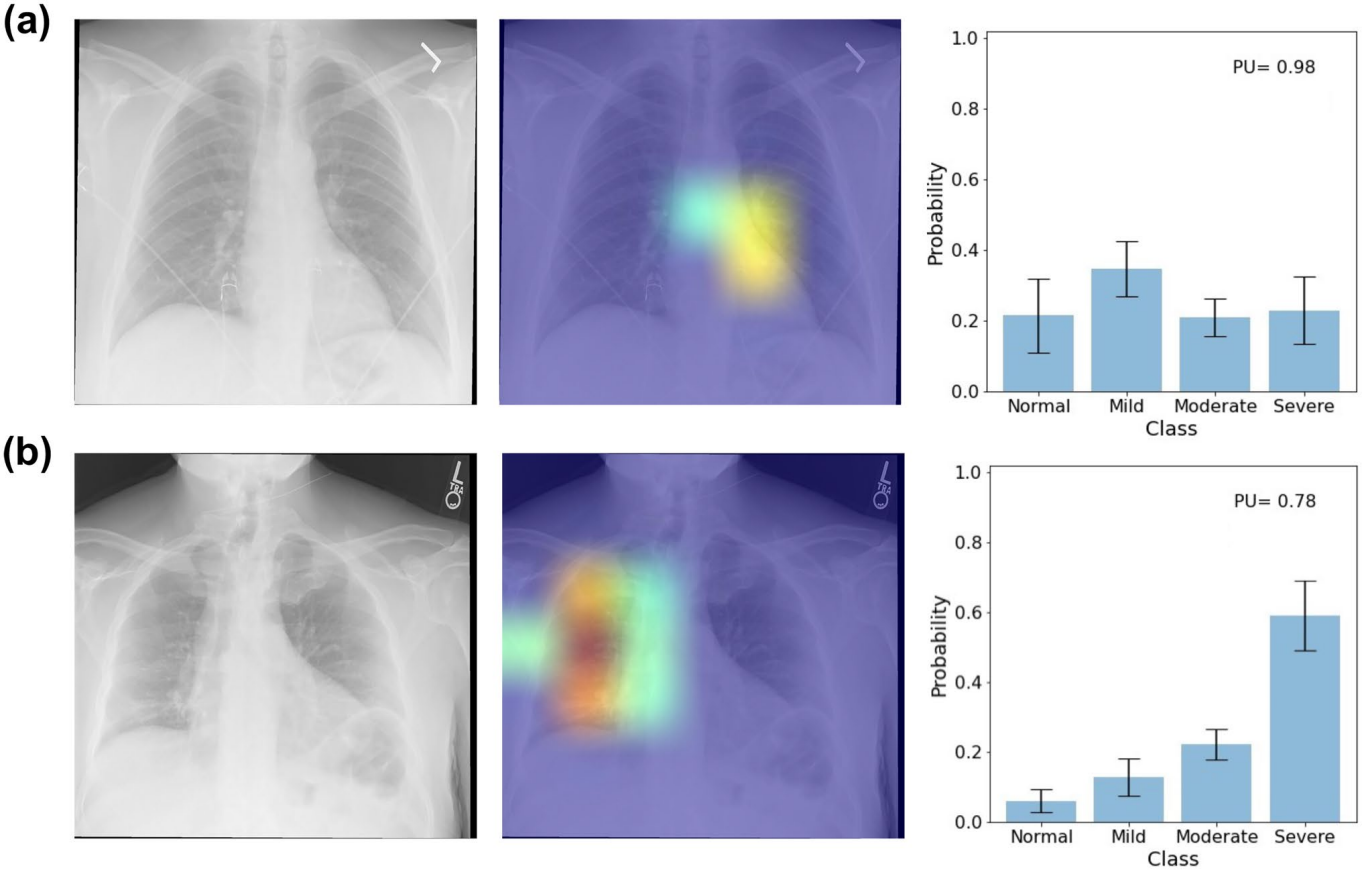
Examples of input CXRs with the windowing issue and the corresponding outputs of the model. The model has very high PU for these inputs (0.98 for (**a**) and 0.78 for (**b**))

The PU of the model was calculated for Dataset 1 over each severity class and presented in Fig. 5(a). The mean PU of the test set was 0.35, 0.55, 0.64, and 0.36 for the normal, mild, moderate, and severe classes, respectively. The mean overall PU was 0.50. Additionally, to compare the uncertainty of the labeling in the model and human readers, we calculated the PU versus the maximum class difference in labeling of the radiologists as shown in Fig. 5b. As presented in Fig. 5, the model had a higher PU for the CXRs from mild and moderate class (Fig. 5a) and had a higher PU over cases that had less agreement among the human readers (Fig. 5(b)). We also tested the model over some low-quality CXRs to evaluate its performance and uncertainty. As shown in Fig. 6, our model provided high uncertainty over the images that had windowing issue.

## Discussion

In this study, we evaluated the performance of a DL model based on BNN approximation through MCD for severity assessment of COVID-19 pneumonia from CXRs on internal and external datasets from two hospitals and compared the results with the readings of human experts. Our results showed that model can predict the severity of the disease with higher performance compared to human experts. We showed that the model had relatively similar performance when tested on external datasets. However, the sensitivity decreases more for the mild and moderate classes, which are more challenging to discriminate.

While DL models have shown promising performance in some medical applications like organ segmentation, their performance and clinical implementation have been problematic due to various issues. As an example, among many developed approaches for diagnosis of COVID-19 from CXRs, there are many few that can be employed in practice [25]. It is also more helpful to use these approaches for severity assessment of the diseases rather than detecting the disease as many test kits are available. One of the main differences of the presented model with prior works is utilizing a confidence-aware model for uncertainty estimation in the prediction and severity assessment of the disease. While some progresses have been made in utilizing BNNs for medical image segmentation and classification [31–33], there are limited works (if any) that considered severity assessment of the disease and compared and evaluated uncertainty with human readers on multi-reader and multi-institute datasets. We believe that it is crucial for any AI-assisted tool in medicine to provide the confidence of the prediction over input samples. To facilitate the usability of PU in clinical readings, this value can be quantized into low, medium, and high uncertainty levels. This information then can be presented in addition to the saliency maps and class probabilities for more reliable human–machine cooperation. In our future study, we will consider the effect of the confidence of the model, and other outputs on the performance of human readers.

It should also be noted that the performance of the AI models highly depends on the data and label quality, and these methods might perform poorly on noisy data. This can become misleading where the prediction of the model is used in clinical studies as an assistant to human readers. BNNs and their approximations can be used to overcome this issue by providing the uncertainty of the prediction over new data. As we showed in the results, the model provided high uncertainty over CXRs with low quality. The model also showed lower certainty in average for the intermediate the intermediate severity classes (i.e., mild and moderate) which are more difficult to discriminate. This agrees with higher inter- and intra-reader variability of human experts over these classes as discussed in [6]. This can be very essential where such tools are being used in clinical environments as an initial assessment and can help the human readers to disregard the AI predictions when the confidence is low.

There are some limitations to this study, which needs further investigation in the future. First, to have a better generalization and performance, the model should be trained on a larger and multi-national dataset that includes a larger variety of patient groups. The other challenge that requires attention is the definition of the reference standard and the ground truth label. In this study, the ground truth labels were considered as the median of the labels of the radiologists. However, high variability in the labeling of the readers might impose some label noise and should be considered [34, 35]. In the future, probabilistic labels instead of one-hot-encoded and hard labels can be used during the training. Additionally, more NN architectures such as ResNet and EfficientNet should be investigated to benchmark their performance for the classification of CXRs. Another extension of this work can be using the BNN for active learning and minimizing the number of required labels from human readers for training the model [27]. The model can be trained on an initial subset of labeled data and additional labels can be requested for the cases where the uncertainty is higher than the maximum acceptable value. This approach can also be extended for classification and diagnosis of other classes of lung diseases.

In conclusion, our results show that BNN approximation through MCD can be a viable solution for severity assessment of lung diseases from CXRs. AI-assisted tools can be used as an adjunct to the radiologists for initial assessment and can facilitate and accelerate the labeling. Finally, the uncertainty measures like PU can be a tool to increase AI transparency and aid clinicians in properly using AI to enhance the accuracy of their diagnosis.

## Data Availability

The datasets employed in this study are available under reasonable request. Please contact Dr. Carlo De Cecco and Dr. Ali Adibi for any request regarding the data.
